# The efficacy of specialised rehabilitation using the Op-reha Guide for cancer patients in palliative care units: protocol of a multicentre, randomised controlled trial (JORTC-RHB02)

**DOI:** 10.1186/s12904-020-00670-6

**Published:** 2020-10-22

**Authors:** Nanako Nishiyama, Yoshinobu Matsuda, Noriko Fujiwara, Keisuke Ariyoshi, Shunsuke Oyamada, Keiichi Narita, Ryouhei Ishii, Satoru Iwase

**Affiliations:** 1 Graduate School of Comprehensive Rehabilitation, Osaka Prefecture University, 3-7-30, Habikino, Habikino-city, Osaka 583-8555 Japan; 2 Department of Clinical Research, NPO JORTC, Tokyo, Japan; 3 Department of Psychosomatic Internal Medicine, National Hospital Organization Kinki-Chuo Chest Medical Centre, Sakai, Japan; 4 IMSUT Hospital of the Institute of Medical Science, The University of Tokyo, Tokyo, Japan; 5 JORTC Data Centre, NPO, Tokyo, Japan; 6 Graduate School of Medicine, Kyoto University, Kyoto, Japan; 7 Department of Emergency & Palliative Medicine, Faculty of Medicine, Saitama Medical University, Saitama, Japan

**Keywords:** Specialised rehabilitation, Palliative care unit, Terminal cancer patient, End of life, Quality of life, Activities of daily living, Randomised controlled trial

## Abstract

**Background:**

Although rehabilitation is recommended for terminal cancer patients, the specific components and methods of such programs are poorly documented. No studies to date have examined the effectiveness of rehabilitation for terminal cancer patients. This study aims to evaluate the efficacy of a new intervention for rehabilitation therapists, using the Op-reha Guide (Guide to Optimal and Patient-Centred Rehabilitation Practice for Patients in Palliative Care Units [PCUs]) in rehabilitation practice. This guide consists of recommended actions and attitudes for rehabilitation therapists and aims to optimise therapists’ actions according to the patient’s needs and condition. It shares goals with terminal cancer patients to maintain their activities of daily living (ADL).

**Methods:**

This study uses a multicentre, prospective, randomised controlled trial (RCT) design with two parallel groups in PCUs where specialised rehabilitation will be routinely performed for terminal cancer patients by rehabilitation therapists. Participants will be randomised (1:1) to intervention (the Op-reha Guide) and control groups (usual rehabilitation). We will then conduct an observational study in PCUs that do not perform specialised rehabilitation for terminal cancer patients; this will be considered the usual care group, and the efficacy of usual rehabilitation will be quantitatively evaluated. Inclusion criteria are hospitalisation in PCU, European Cooperative Oncology Group Performance Status of 2 or 3, and clinical estimation of life expectancy of 3 weeks or more. Patients with severe symptom burden will be excluded. We hypothesise that the Op-reha Guide will be more effective in maintaining the ADL of terminal cancer patients hospitalised in PCUs than usual rehabilitation. The primary endpoint is defined as the change in (total) modified Barthel Index from baseline to Day 22. Quality of life will be a secondary endpoint. In total, 135 patients will be recruited from 16 Japanese sites between July 2019 and December 2021.

**Discussion:**

This will be the first trial to evaluate the efficacy of specialised rehabilitation for terminal cancer patients hospitalised in PCUs, and will contribute to the evidence on the efficacy of implementing rehabilitation for terminal cancer patients.

**Trial registration:**

*UMIN-CTR,* UMIN000037298 R000042525 (date of registration 7 July 2019).

## Background

Cancer patients in the terminal phase experience a decline in physical function and activities of daily living (ADL). ADL decline occurs in many cancer patients between 1 and 3 months before death [1, 2]. Many cancer patients wish to maintain their independence even in the terminal phase; thus, the deterioration of physical function and ADL in terminal cancer patients may cause a decrease in their quality of life (QOL) [3, 4]. This is a significant issue for these patients, their families, and healthcare professionals.

In recent years, opportunities for providing rehabilitation to cancer patients have expanded [5]. Cancer rehabilitation is a medical approach that aims to improve cancer patients’ QOL [6] by helping them maintain maximum physical, social, psychological, and vocational functioning within the limits imposed by the disease and its treatment [7]. At the same time, clinical studies on rehabilitation for cancer patients have also increased. These studies have found, for example, that physical exercise for cancer patients has beneficial effects, including improved physical function and reduced symptom burdens [8–19]. However, most participants in these studies were in a disease phase where they could recover their physical function and ADL, such as early mobilisation for perioperative patients, therapeutic exercise during cancer treatments, and physical fitness for cancer survivors, with patients’ prognosis being about 3 to 12 months. In contrast, there are a few studies on rehabilitation that have focused on terminal cancer patients. Moreover, these previous studies in terminal cancer patients were limited to single case reports and surveys on actual situations [20–23]. These studies in terminal cancer patients reported the actual situation of rehabilitation for patients in hospices or palliative care units (PCUs), such as the amount of rehabilitation provided and the content of the implemented rehabilitation programs; they did not exclusively focus on the patients with terminal cancer. More than half the patients in these studies of terminal cancer patients were either alive or had been discharged at the end of the study period. Thus, there were no studies that focused exclusively on patients in the terminal phase.

In other words, although it is recommended that rehabilitation be performed for terminal cancer patients, even during the last days of life [23–25], the effectiveness of rehabilitation for terminal cancer patients has not been evaluated. Furthermore, the specific content and methods of rehabilitation interventions, such as how often, how long, and what kind of rehabilitation should be performed for these patients, are poorly documented. Therefore, rehabilitation therapists currently deliver treatment to patients based on their own experiences; this may cause differences in the quality of rehabilitation provided [26, 27]. In addition, as many cancer patients become increasingly frail in the terminal phase [1, 25], routine interventions might be harmful. Thus, methodologically rigorous studies for the best rehabilitation interventions for terminal cancer patients are urgently needed.

The aim of this study is to evaluate the efficacy of a new, comprehensive guide for rehabilitation therapists, the “Guide to Optimal and Patient-Centred Rehabilitation Practice for Patients in PCUs” (Op-reha Guide), designed to maintain ADL of terminal cancer patients hospitalised in PCUs. An intervention group will receive treatment based on the Op-reha Guide while a control group will receive usual rehabilitation. The study may contribute evidence on the efficacy of implementing rehabilitation with this guide for terminal cancer patients in PCUs.

## Methods

### Design

**Fig. 1:**
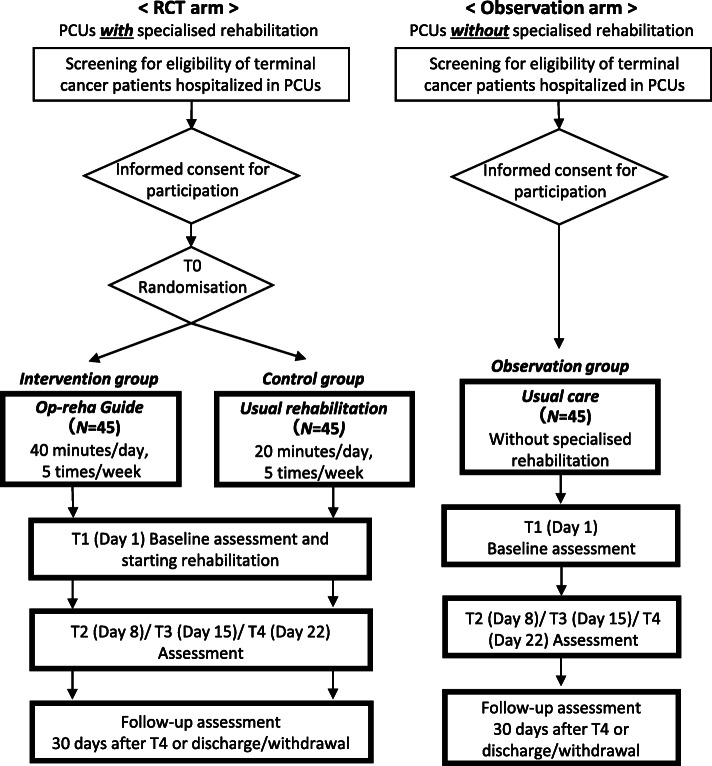
Flow diagram of the study procedure. Participants will be randomized (1:1 allocation ratio) to Op-reha Guide (intervention group) or usual rehabilitation (control group) in the RCT arm. Assessments will be performed at baseline (T0 randomisation), day 1 (T1), day 8 (T2), day 15 (T3), and day 22 (T4) in RCT and observation arms. Notes. RCT = randomised controlled trial, T = time of assessment, PCU = palliative care unit

This study uses a multicentre, prospective, randomised controlled trial (RCT) design with two parallel groups as the RCT arm, and will conduct an additional observational study with a single observational group (usual care) as the observation arm. The study design is summarised in Fig. 1.

In our study, specialised rehabilitation is defined as the treatment provided by rehabilitation therapists, occupational therapists, and physiotherapists. The RCT arm will be conducted in PCUs that routinely perform specialised rehabilitation for terminal cancer patients. The participants of the RCT arm will be randomised (1:1) to the intervention group (the Op-reha Guide) and the control group (usual rehabilitation). The observation group will come from PCUs that do not routinely have rehabilitation therapists perform rehabilitation for terminal cancer patients. The control group will be observed in PCUs where only usual rehabilitation for terminal cancer patients occurs. Observation of the intervention group will occur in PCUs that perform rehabilitation based on the Op-reha Guide. Subsequently, we will compare the patients in the intervention group to those in the control group to discover the effects of using the Op-reha Guide versus usual rehabilitation. We will compare the control group with the observation group to quantitatively evaluate the efficacy of usual rehabilitation. The protocol of this study followed the Standard Protocol Items Recommendations for Interventional Trials (SPIRIT) statement [28] and its checklist.

### Participants and setting

Participants will be recruited from 16 PCUs across Japan. There will be 11 PCUs in the RCT arm and 5 PCUs in the observation arm, and participants will be enrolled according to the inclusion and exclusion criteria. Inclusion criteria are: a) an initial hospitalisation in PCU; b) being 20 years or older at the time when they provide informed consent; c) having a clinical diagnosis of cancer; d) receiving no curative treatment, such as surgery, radiation therapy, or chemotherapy; e) European Cooperative Oncology Group Performance Status (ECOG PS) of 2 or 3; f) having a clinically estimated life expectancy of 3 weeks or more (based on the opinion of investigators and Palliative Prognostic Index of 6 or less); g) starting specialised rehabilitation within 1 week of hospitalisation in PCU (only RCT arm); and h) having the ability to provide written informed consent. Exclusion criteria are: a) having a severe symptom burden (i.e. score of 4 on any of the three Support Team Assessment Schedule items of pain control, other symptom control, and patient anxiety); b) having a respite admission within 1–2 weeks; c) having a weight-bearing limitation due to impending bone fracture; and d) will be undergoing a nerve block anaesthesia or percutaneous vertebroplasty (cement composition).

### Measurement tools

#### Modified Barthel Index (mBI)

The mBI [29] is a modified version of the Barthel Index (BI) and uses a 5-point scale, which is more sensitive to changes in the ability to perform ADL than the BI [30]. The BI assesses 10 daily tasks: personal hygiene, feeding, bathing self, dressing, toilet, bowel control, bladder control, chair/bed transfers, ambulation, and stair climbing. The mBI reflects the level of independence, from 0 (*needs full assistance*) to 100 (*independence*). The reliability and validity of the Japanese version of mBI have been confirmed [31, 32].

#### European Cooperative Oncology Group Performance Status (ECOG PS)

The ECOG PS includes six grades, from 0 (*fully active*) to 5 (*died*), to assess how the disease affects the daily living abilities of the patient [33].

#### Palliative Performance Scale (PPS)

The PPS comprises 11 levels, from 0% (*death*) to 100% (*full*), to assess a patient’s ambulation, activity and evidence of disease, self-care, intake and consciousness level [34, 35]. The PPS is also used as a prognostic tool to estimate the survival in palliative care patients [34, 36].

#### Palliative Prognostic Index (PPI)

The PPI uses PPS and four bedside parameters to predict a probabilistic short-term survival of terminally ill cancer patients, from 0 to 15 (i.e. likelihood of being alive at 3 and 6 weeks). A total score over 6 predicts a survival of less than 3 weeks [37, 38]. The PPI may be sufficient to evaluate prognosis in cases where laboratory values are not available [39]. Limitations of the PPI include the difficulty of accurately diagnosing delirium and its low negative predictive value.

#### Support Team Assessment Schedule (STAS)

STAS provides a score from 0 (*best*) to 4 (*worst*) to assess a patient’s symptom burden [40]. The reliability, validity and inter-rater reliability of the Japanese version have been confirmed [41, 42].

#### European Organisation for Research and Treatment of Cancer (EORTC) Quality of Life Questionnaire Core 15 Palliative care (QLQ-C15-PAL)

The EORTC QLQ-C15-PAL will be used to assess the patient’s QOL [43]. The EORTC QLQ-C15-PAL is a short version of the EORTC QLQ-C30; it consists of 15 items as a core questionnaire for palliative care settings. The global QOL item is rated from 1 (*very poor*) to 7 (*excellent*), and the others are rated from 1 (*not at all)* to 4 (*very much*). The reliability and validity of the Japanese version have been confirmed [44].

### Procedures

#### Enrolment and allocation

Patients hospitalised in PCUs will be screened for eligibility. Eligible patients will be informed of the study procedure, data protection plan, risks, and benefits by investigators. In the RCT arm, each patient who agrees to participate and provides written consent will be randomly allocated to either the intervention group (Op-reha Guide) or the control group (usual rehabilitation). The patients in the RCT arm will start rehabilitation within 7 days after hospitalisation. In the observation group, each patient who agrees to participate and provides written consent will start observation within 7 days of hospitalisation; they will not be randomly allocated.

Data on eligible patients who have given informed consent will be collected and managed using a web-based electronic data capture system in the Japanese Organisation for Research and Treatment of Cancer (JORTC) Data Centre. We used a computer-generated randomisation schedule using stratified permuted block randomisation methods to assign patients to either the intervention or control group in a 1:1 ratio, balanced for the following stratification factors: 1) ECOG PS is 2 or 3; and 2) study site.

#### Blinding

This study follows a single-blind design. In the RCT arm, we will not provide information to patients as to which of the two protocol treatments is based on the Op-reha Guide and which is based on usual rehabilitation. Hence, patients will be blinded to the intervention, but the therapists and other collaborators will be unblinded.

#### Data collection

Assessments will be performed at five time points: enrolment (T0 eligibility and randomisation), Day 1 (T1 baseline), Day 8 (T2), Day 15 (T3), and Day 22 (T4) by investigators consistently at each site. The Day 1 assessment is performed on the day rehabilitation begins in the RCT arm, and on the hospitalisation day within 7 days in the observation group. If a patient is discharged or withdraws before T4, a withdrawal assessment will be performed. If a patient continues to be hospitalised after withdrawal, the remaining measurements will be performed according to the assessment schedule. The follow-up assessment is performed 30 days after T4 or withdrawal. The timing and details of assessments are given in Table 1.

**Table 1:** Study procedure and time points for actions and evaluations

	T0 Enrolment	T1 Day1 Baseline	T2 Day 8	T3 Day 15	T4 Day 22	Discharge or Withdrawal	Follow-up30 days after T4 or discharge/ withdrawal
Demographics		x	x	x	x	x	
mBI		x	x	x	x	x	
EORTC QLQ-C15-PAL		x	x	x	x	x	
ECOG PS	x	x	x	x	x	x	
PPS		x	x	x	x	x	
STAS	x	x	x	x	x	x	
PPI	x	x					
Adverse events (RCT)		x	x	x	x	x	
Details of the rehabilitation (RCT)			x	x	x	x	
Reason for stopping (RCT)						x	
Clinical outcome							x

*mBI* modified Barthel Index, *EORTC QLQ-C15-PAL* European Organisation for Research and Treatment of Cancer Quality of Life Questionnaire Care 15 Palliative Care, *ECOG PS* European Cooperative Oncology Group Performance Status, *PPS* Palliative Performance Scale, *STAS* Support Team Assessment Schedule, *PPI* Palliative Prognostic Index, *RCT* Randomised Controlled Trial

### Interventions

The specialised rehabilitation according to the Op-reha Guide and usual rehabilitation will be administered for 3 weeks in the RCT arm by rehabilitation therapists. After the study is completed or the patient withdraws, rehabilitation and care according to this study protocol will end, and the patient will receive the site’s usual treatment. As study participation is voluntary, the patients can any time withdraw from the study.

#### Op-reha Guide

The Op-reha Guide is a newly developed intervention guide for rehabilitation therapists designed to address the need to maintain the ADL of terminal cancer patients hospitalised in PCUs. It was developed by integrating both the experience of clinical practice and the literature review by the multidisciplinary team. The team consisted of a rehabilitation therapist (OT and PT), a nurse, a clinical psychologist, and three physicians specialising in palliative care. We also included the opinions of other rehabilitation therapists with expertise in palliative care. The core concepts of the Op-reha Guide are: 1) to optimise the actions and attitude of rehabilitation therapists according to the patient’s needs and condition; 2) to share the goals with the patient; 3) to implement rehabilitation designed to maintain ADL. The Op-reha Guide consists of 22 recommended actions in total; the basic length is 40 min per day, with a frequency of five times per week to adequately perform the actions.

To increase adherence to the protocol intervention and reduce the risk of contamination, we take some measures with the operation method of the Op-reha Guide as follows. First, we will prepare a detailed operational procedure manual and explain the manual in detail for the site investigators at the kick-off meeting, instead of providing training. Only the therapist in charge of patients allocated to the intervention group will be able to access the guide and only for the duration of their intervention. Next, the therapist will receive a recommended list of set actions for each implementation day, with a checklist that must be filled out after each treatment. The therapists may treat both intervention and control group patients but cannot be in charge of the two groups at the same time.

#### Usual rehabilitation as control

Currently, there is no specific standard rehabilitation for terminal cancer patients hospitalised PCU in Japan. Thus, we defined the basic length as 20 min per day, five times per week, which is the average rehabilitation length and frequency for cancer patients hospitalised PCUs in Japan according to the most frequent response in a questionnaire survey conducted in Japan [45].

#### Usual care (observation group)

The observation group will receive usual care without specialised rehabilitation during hospitalisation. Usual care will be provided to all patients by physicians, nurses, and allied health professionals without rehabilitation therapists.

### Study endpoints

We hypothesise that the intervention group will more effectively maintain their ADL than the control group. Therefore, we selected ADL as the primary endpoint and chose QOL as one of the secondary endpoints.

The primary endpoint is the change in total mBI from baseline to T4. The secondary endpoints are as follows: 1) change in total mBI from baseline to T3/T2; 2) change in each sub-item score of mBI from baseline to T4/T3/T2; 3) longitudinal change in total and sub-item scores of mBI at baseline, T2, T3, and T4; 4) proportion of patients who show a decrease of 10 points or more in total mBI from baseline to T4/T3/T2; 5) change in each domain score of EORTC QLQ-C15-PAL from baseline to T4/T3/T2; and 6) adverse events in the RCT arm.

### Analysis

#### Data management and monitoring

All data will be collected by the JORTC Data Centre, which will also oversee the intra-study data sharing process. The clinical data entry, data management, and central monitoring will be conducted using the electronic data capture system REDCap (developed by Vanderbilt University, USA).

#### Study population for analyses

The population for efficacy and safety analyses comprises all patients who receive at least one session of the protocol intervention. Patients who are found to be ineligible after registration will be excluded from the efficacy analysis, although they will be included in the safety analysis (adverse events).

#### Statistical analysis

Comparison of the primary endpoint (i.e. mean difference in the change in total mBI from baseline to T4) between the intervention group and control group will be conducted using a two-sided one-way *t*-test with a significance level of 5% according to the intention-to-treat principle. Point estimates and 95% confidence intervals for the mean difference between the two groups will be calculated. This analysis will also be conducted between the control group and observation group. For supplementary analysis that compares the control and observation groups, a multivariate analysis will be conducted with the primary endpoint as the dependent variable, and groups and background information as independent variables. The secondary endpoints of efficacy will be evaluated similarly. For the safety evaluation, the frequency and incidence of adverse events for each group will be calculated. A full statistical analysis plan will be written prior to the data evaluation. All analyses will be performed using SAS version 9.4 (SAS Institute, Cary, NC, USA).

#### Sample size calculation

To calculate the sample size for the RCT arm, we assumed that the mean difference in the change in total mBI from baseline to T4 would be 10 (standard deviation, 15) between the groups. There was no previous study on the minimal clinically significant difference (MCID) in mBI at the planning stage of this study. Thus, we decided to adopt a 10-point difference compared with usual rehabilitation as the MCID of this study by extrapolating the MCID of the 20-point scale of the BI [46]. Assuming a 15–20% attrition rate, we calculated 90 patients (45 patients in each group) with a two-sided significance level of 5% and a power of 80%. We decided that the sample size of the observation group would also be 45 patients.

## Discussion

Our review of the existing literature found that there has been no randomised study that evaluates the efficacy of specialised rehabilitation for terminal cancer patients hospitalised in PCUs. In this study, we will adopt a multicentre, randomised controlled trial design. This is the most appropriate study design to evaluate the efficacy of a new approach. We also considered a cluster randomised controlled design, but chose an individually randomised controlled design due to the exclusion features of institutions, and expect demographic characteristics and other possible confounders to be adjusted for and the results to be generalizable [47]. In addition, we will conduct an observational study, allowing us to evaluate the efficacy of the control group (usual rehabilitation) quantitatively compared with the observation group (usual care).

We would like to discuss several issues related to practice, operation, and other aspects of the study. First, the risks of contamination from the intervention group to the control group are inevitable because it is not possible to completely separate the therapists providing interventions based on the Op-reha Guide and usual rehabilitation. This means that the efficacy of the guide versus the therapist may be difficult to evaluate. To reduce the risk of contamination, we have taken some measures with operating procedures; the therapists cannot be in charge of the two groups at the same time; only the therapist in charge of patients allocated to the intervention group will be able to access the guide and only for the duration of their intervention.

Second, it is assumed that the therapists cooperating with this study are biased towards being highly motivated and experienced regarding rehabilitation in palliative care units. This may also reduce a difference in efficacy between the intervention and control group.

Third, due to ethical concerns, we did not randomise three groups as a whole as this could cause differing participant characteristics between the RCT arm and the observation arm. Since the efficacy of usual rehabilitation on terminal cancer patients has not been examined previously, we need to compare the usual rehabilitation with a group that does not receive any additional specialised rehabilitation as a usual care group. Because we could not ignore the possibility that patients assigned to the observation group might require the specialised rehabilitation, we conducted the observation group in PCUs that not routinely performed specialised rehabilitation.

Furthermore, the patients targeted in this study are in the terminal phase and have a short life expectancy, the risk of withdrawal might be higher than the patients who are not in the terminal phase. It also means that the risk of being unable to adhere to the prescribed intensity, frequency, and time of the protocol intervention may be higher. However, this does reflect the actual status of clinical practice of rehabilitation for terminal cancer patients who are hospitalised in PCUs.

Since this study is the first of its kind, it will contribute to the evidence on efficacy of implementing rehabilitation for terminal cancer patients.

### Status of the trial

Enrolment started in July 2019. At the time of manuscript submission (July 2020), a quarter of the patients have participated. Thus, we expect to complete recruitment by December 2021.

## Data Availability

Not applicable. The JORTC Data Centre and JORTC Independent Data Monitoring Committee have access to the final dataset of this trial. There is no contractual agreement with regards to investigators’ access restrictions on the dataset.

## Abbreviations

RCT: Randomised controlled trial
ADL: Activities of daily living
QOL: Quality of life
mBI: Modified Barthel Index
EORTC: European Organisation for Research and Treatment of Cancer
QLQ-C15-PAL: Quality of Life Questionnaire Core 15 Palliative care
ECOG PS: European Cooperative Oncology Group Performance Status
PPS: Palliative Performance Scale
STAS: Support Team Assessment Schedule
PPI: Palliative Prognostic Index
MCID: Minimal Clinically Significant Difference
